# Adaptive Global Power-of-Two Ternary Quantization Algorithm Based on Unfixed Boundary Thresholds

**DOI:** 10.3390/s24010181

**Published:** 2023-12-28

**Authors:** Xuefu Sui, Qunbo Lv, Changjun Ke, Mingshan Li, Mingjin Zhuang, Haiyang Yu, Zheng Tan

**Affiliations:** 1 Aerospace Information Research Institute, Chinese Academy of Sciences, No. 9 Dengzhuang South Road, Haidian District, Beijing 100094, China; suixuefu19@mails.ucas.ac.cn (X.S.); lvqb@aircas.ac.cn (Q.L.); kecj@aircas.ac.cn (C.K.); lims@aircas.ac.cn (M.L.); zhuangmj@aircas.ac.cn (M.Z.); yuhy@aircas.ac.cn (H.Y.); 2 Department of Key Laboratory of Computational Optical Imagine Technology, Chinese Academy of Sciences, No. 9 Dengzhuang South Road, Haidian District, Beijing 100094, China

**Keywords:** convolutional neural network, power of two, unfixed boundary thresholds, ternary quantization, low accuracy loss

## Abstract

In the field of edge computing, quantizing convolutional neural networks (CNNs) using extremely low bit widths can significantly alleviate the associated storage and computational burdens in embedded hardware, thereby improving computational efficiency. However, such quantization also presents a challenge related to substantial decreases in detection accuracy. This paper proposes an innovative method, called Adaptive Global Power-of-Two Ternary Quantization Based on Unfixed Boundary Thresholds (APTQ). APTQ achieves adaptive quantization by quantizing each filter into two binary subfilters represented as power-of-two values, thereby addressing the accuracy degradation caused by a lack of expression ability of low-bit-width weight values and the contradiction between fixed quantization boundaries and the uneven actual weight distribution. It effectively reduces the accuracy loss while at the same time presenting strong hardware-friendly characteristics because of the power-of-two quantization. This paper extends the APTQ algorithm to propose the APQ quantization algorithm, which can adapt to arbitrary quantization bit widths. Furthermore, this paper designs dedicated edge deployment convolutional computation modules for the obtained quantized models. Through quantization comparison experiments with multiple commonly used CNN models utilized on the CIFAR10, CIFAR100, and Mini-ImageNet data sets, it is verified that the APTQ and APQ algorithms possess better accuracy performance than most state-of-the-art quantization algorithms and can achieve results with very low accuracy loss in certain CNNs (e.g., the accuracy loss of the APTQ ternary ResNet-56 model on CIFAR10 is 0.13%). The dedicated convolutional computation modules enable the corresponding quantized models to occupy fewer on-chip hardware resources in edge chips, thereby effectively improving computational efficiency. This adaptive CNN quantization method, combined with the power-of-two quantization results, strikes a balance between the quantization accuracy performance and deployment efficiency in embedded hardware. As such, valuable insights for the industrial edge computing domain can be gained.

## 1. Introduction

### 1.1. Background

Since the 21st century, with the increasing number of real-time application scenarios [1,2,3,4] such as drones, the Internet of Things (IoTs), intelligent cars, and satellite data in-orbit processing, which require the data generated by different sensors to be processed quickly, the field of edge computing has developed rapidly, which can reduce the transmission delay of data and improve the real-time performance by completing the relevant processing of the data directly in embedded chips [5,6]. Furthermore, deep convolutional neural networks (DCNNs) are the most crucial intelligent data processing algorithms in the field of artificial intelligence [7,8,9]. Leveraging their powerful capabilities for representation learning and information perception, relevant industries have been exploring the extensive applications of convolutional neural networks in edge computing scenarios with the aim of achieving more efficient data processing [10,11,12,13]. However, the superior performance of CNNs relies on their large number of parameters, thereby leading to high computation volumes. For instance, VGG-16 [7] consists of 135 million parameters and requires 30.8 billion calculations to complete a single image detection task on a 224 × 224 pixel image. Such demands necessitate hardware with abundant computational and storage resources, such as GPUs (e.g., Nvidia 4090Ti and Nvidia A100) [14], to provide the necessary computing support. However, conventional embedded chips (e.g., FPGAs and ASICs) provide fewer on-chip computation and storage resources due to their limited size, energy consumption, and external heat dissipation conditions [15,16]. The edge deployment of CNN models often requires external memory like Double Data Rate SDRAM (DDR) due to the large number of parameters. This necessitates frequent reads from external memory during the forward inference process, thus increasing deployment complexity. Moreover, due to the transmission delay of the external memory, the data supply to the computation unit may not be timely, thereby leading to a mismatch between the data reading rate and the computing rate, which thus affects the computing efficiency and system performance of the convolution computation module in the chip [17,18]. At the same time, the large number of high-bit-width floating-point multiplication and addition calculations will lead to insufficient computing resources, thereby increasing the computing time and energy consumption. Therefore, it is necessary to reduce the calculation parallelism in the chip or perform low-precision calculations by cutting parameters during the computation process in order to reduce the pressure on the computation and bandwidth in the chip and ensure the normal embedded operation of CNN algorithms [10,19]. These issues are currently the main factors that make it challenging for CNN algorithms to achieve both real-time and high-accuracy performance in industrial edge computing applications [4,20,21].

### 1.2. Existing Methods and Problems

In all edge computing chips (e.g., CPU, GPU, FPGA, and ASIC), reducing memory and increasing parallelism is the fundamental solution to the problem of the poor real-time performance of CNNs [22,23,24]. Therefore, the compression of CNN models is the most meaningful research field for the application of CNNs in the edge computing field. At present, mainstream research directions mainly include new lightweight CNN architecture design [25,26], model pruning [27,28], low-rank matrix factorization [29], and parameter quantization [30,31,32,33,34]. Among them, the parameter quantization method maps the network weights from 32-bit floating points to low-bit-width fixed points, thus converting the network computing mode to low-bit-width fixed-point computing, which can effectively save hardware bandwidth and energy consumption, reduce storage space, and increase computing parallelism in theory. It is suitable for improving computational efficiency in edge computing chips with few resources [23]. Whether adopting a new architecture design or conducting model pruning optimization, it is necessary to quantize the weights in new models. This is the main reason why many edge applications use parameter quantization as the preferred method of algorithm optimization [35,36]. Generally, the reduction in bit width weight can lead to a higher compression ratio and faster processing speed in the hardware; however, at the same time, the expressive ability of the weight is also reduced, thereby decreasing the accuracy of the CNN accordingly. To date, many studies have proved that there is high redundancy in 32-bit floating-point CNN models, and 8-bit quantization can achieve quantization without accuracy loss in most CNNs [36,37,38]. However, lower-bit quantization often leads to unacceptable accuracy loss, especially in quantization studies with extremely low bit widths, such as below 3-bit [31,32,39,40,41]. Courbariaux et al. [31] and Rastegari et al. [32] successively proposed the ultimate 1-bit quantization method: a binary weight approximate neural network and XNOR-Net that quantize weights to −1 and +1 and can use addition or subtraction operations and bitwise operations to replace the original multiplication calculations in convolutional neural networks. Both studies compressed the model storage by 32× and achieved a 58× forward inference increase in the CPU platform. However, the accuracy losses of the two algorithms both exceeded 10%. In order to balance the quantization accuracy and hardware performance of binary CNNs, Li et al. [40] proposed a ternary (2-bit) CNN quantization method called TWN. By adding a value of 0 on the basis of binary quantization, the TWN quantized the weights into three values {−1,0,1}, which improved the expressive ability of the model. In small classification data sets such as MNIST [42], it achieved an accuracy similar to that of the baseline network, while, in medium and large data sets, the accuracy loss was still significant and maintained at around 4%. Later, Zhu et al. [41] proposed a more general ternary quantization method, called TTQ, to obtain a higher accuracy. This approach is not limited to the quantization of values to ±1 but quantizes the weight of each layer to zero and the other two positive and negative 32-bit values $\left\{{-W}_{l}^{n},\;0,\;W_{l}^{p}\right\}$. The latest research on ternary quantization, such as those introducing the LCQ [34] and RTN [43] algorithms, has controlled the ternary quantization accuracy loss to within 2%, thereby achieving excellent performance at the software level. However, the abovementioned studies, as well as most other low-bit quantization studies [44,45], all involved nonglobal quantization, which leads the obtained quantization models to retain some 32-bit floating-point weights without quantization. If all weights were fully quantized, the accuracy loss would be unacceptable. This poses significant challenges for deploying these quantized CNN models onto embedded chips. Similar to the original 32-bit models, they still require substantial storage and computational resources, thereby resulting in a lack of computational efficiency and lower practicality.

In our previous study [46], in order to solve the problem of nonglobal quantization, we proposed a fine-grained global low-bit quantization architecture that can achieve quantization with no accuracy loss compared to the 32-bit baseline models, at 3-bit bit widths and above while quantizing all weights in the network. However, in the case of binary quantization (2-bit), although our previous study achieved better performance than most other quantization algorithms, the accuracy loss was still around 2% compared to the 32-bit baseline models. It can be seen that there are currently few methods that can achieve minimal accuracy loss in global ternary quantization. Therefore, studying how to further approach the accuracy of the baseline model while carrying out global ternary quantization is the focus of our research. Some previous research has treated parameter quantization as an optimization problem, such as studies involving TWN and TTQ approaches [40,41,47]. These approaches determine the quantization scaling factor by calculating the minimum Euclidean distance (i.e., L2 norm) between the original floating-point weights and the corresponding quantization weights, while Nvidia [48] determined the quantization scaling factor by calculating the minimum KL divergence between them. On the other hand, more studies have set quantization ranges and values based on demand directly [34,46] or have linearly quantized the network according to the following formula [35,36,49,50,51]:

(1) $$S\;=\frac{w_{\max}-w_{\min}}{q_{\max}-{\;q}_{\min}},$$

(2) $$Z\;=q_{\max}-\frac{w_{\min}}{S},$$

(3) $$q=\mathrm{clip}\;(\mathrm{round}\;(\frac{w}{S}+Z),\;0,\;2^{n}-1),$$

where *w* represents the unquantized floating-point weights, *q* represents the quantized fixed-point weights, *S* represents the quantization normalization relation (the quantization scaling factor), *Z* represents the quantized fixed-point value corresponding to the 0 value of the unquantized floating-point numbers, clip (·) denotes the boundary clipping function, round (·) denotes rounding to the nearest calculation, and *n* indicates the quantization bit width.

**Figure 1:**
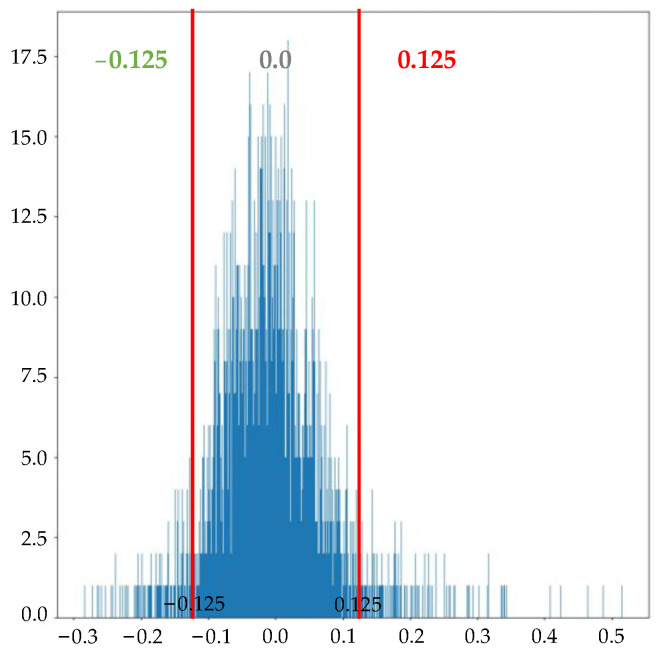
Ternary quantization based on fixed thresholds.

However, regardless of the kind of quantization method adopted, the fundamental essence of current quantization studies is to partition the floating-point weights within each layer based on their actual distribution and to set several fixed boundary thresholds $\theta_{i}$ for each layer. This process creates multiple fixed intervals according to these thresholds, with each interval corresponding to a fixed-point quantization value. This means that weights near the boundary threshold and weights near the corresponding interval center are quantized to the same value. Due to the absence of predetermined patterns in the weight distribution of CNN models, especially concerning fine details [52], if there are massive weights near the fixed boundary threshold, this situation may result in some irrationality, which will lead to certain quantization errors [50,53]. For ternary quantization, Figure 1 shows the weight distribution of a convolutional layer. The red line represents two fixed quantization boundary thresholds. Normal quantization methods quantize the weights as {−0.125, 0, +0.125} based on two red lines such that all weights between −0.125 and 0.125 are quantized; for example, a 0.124 weight is theoretically closer to 0.125, and the local error quantized as 0.125 will be smaller. However, in reality, it is quantified as 0. Furthermore, the two boundary thresholds for ternary quantization are often set in the area with the highest weight in that layer, which can amplify the error caused by fixed boundary thresholds.

In addition, for the deployment of quantized models, Abhinav et al. [54] applied parameter quantization to pathological image analysis and validated it on a Raspberry Pi chip, thereby demonstrating that an 8-bit quantized model, under the same deployment architecture, could improve computational efficiency by 30% compared to the baseline model. Meng et al. [55] verified on an FPGA that, even with comparable resource utilization, high-bit-width models, even at higher operating frequencies, exhibited significantly lower computational efficiency than models with 4 bits or fewer. Li et al. [56] also validated on an Intel CPU that a 2-bit quantized model could achieve more than a 4× improvement in computational efficiency compared to the baseline model. These studies provide compelling evidence for the advantages of parameter quantization in the edge applications of CNNs.

However, some more extreme quantization deployment efforts have shown that, due to the enormous computational workload in CNNs, the scarce on-chip DSP computing units used for multiplication calculations are often fully utilized, thereby significantly increasing the computational and power pressure on the chip [17,57,58]. Moreover, the actual computational efficiency of on-chip DSP units is approximately one-fourth that of other on-chip computing resources [57]. It is evident that the on-chip DSP resources greatly limit the computational efficiency of CNNs in edge deployment. Power-of-two quantization methods such as INQ [33] and APoT [59] involve quantizing all the CNN weights to powers of two or zero. In the binary computation process on edge chips, the multiplication calculations of power-of-two values can be replaced by shift calculations [46]. Since edge chips often adopt a register architecture, this allows for the rapid and cost-effective implementation of shift calculations. This approach can achieve the deployment of quantized networks with fewer on-chip hardware resources, thereby alleviating the limitation imposed by the number of on-chip DSP multipliers on computational efficiency. However, all current power-of-two quantization algorithms are still based on fixed quantization boundary thresholds, thereby preventing the attainment of minimal accuracy loss in 2-bit quantization.

### 1.3. Contributions of This Paper

In order to avoid the constraint of fixed quantization boundary thresholds and enable quantized CNN models with both high accuracy and strong hardware adaptability, this paper focuses on a novel quantization strategy using unfixed boundary thresholds. Research was conducted on the global power-of-two ternary CNN quantization algorithm, and we propose an effective method: the Adaptive Global Power-of-Two Ternary Quantization Algorithm Based on Unfixed Boundary Thresholds (APTQ). The APTQ algorithm introduces a novel adaptive quantization strategy, which, based on the principle of minimizing Euclidean distance, divides the process of directly quantizing weights into power-of-two ternary values, which are divided into two groups of binary quantization processes. This approach aims to achieve nonfixed quantization boundary thresholds, minimize spatial differences between the CNN model before and after quantization, and ensure that the quantized model exhibits hardware-friendly characteristics. At the same time, the APTQ algorithm improves the overall quantization framework on the basis of the fine-grained layered-grouping iteration global quantization framework proposed in the GSNQ algorithm, and it adopts a fine-grained method of layer-by-layer grouping quantization and retraining iterations adapted to the new quantization strategy to complete global CNN quantization and to realize global power-of-two ternary quantization with unfixed boundary thresholds.

Furthermore, the quantization method proposed in this paper based on unfixed boundary thresholds is not limited to ternary quantization, but it can be extended to arbitrary bit width quantization. Based on APTQ, we propose the Adaptive Power-of-Two Quantization algorithm (APT) with wider applicability, which adaptively determines all quantization thresholds and can improve the performance of power-of-two quantization to a certain extent. In summary, this paper builds upon the excellent hardware performance of existing global power-of-two quantization algorithms and further optimizes their software performance. It provides valuable insights and references for the embedded application of large-scale CNNs in the field of edge computing. The main contributions of this paper are as follows:

1. This paper analyzes the nonglobal quantization and fixed quantization threshold problems in existing ternary quantization methods and formulates a new power-of-two ternary quantization strategy with unfixed boundary thresholds based on the global CNN quantization architecture proposed in our previous study [46]. This new quantization strategy decomposes each filter in a CNN model into two subfilters. By minimizing the Euclidean distance, the two subfilters are binarized into a power-of-two form. According to the matrix additivity, the two binary filters are combined into one ternary filter to complete the power-of-two CNN ternary quantization, and the restrictions on CNN performance due to fixed boundary thresholds and intervals are removed.
2. This paper formulates a general power-of-two quantization strategy based on unfixed thresholds. By decomposing each filter into multiple filters and performing binarization and accumulation, the power-of-two ternary quantization strategy with unfixed thresholds can be extended to any bit width quantization.
3. Ternary and other bit width quantization experiments were conducted on mainstream CNN models, such as VGG-16 [7], ResNet-18, ResNet-20, ResNet-56 [60], and GoogLeNet [61], in two image classification data sets: CIFAR10 [62] and Mini-ImageNet [63]. The results were compared and evaluated quantitatively and qualitatively with some state-of-the-art algorithms in order to verify the effectiveness and versatility of the proposed APTQ and APQ algorithms.

The remaining parts of the paper are structured as follows: Section 2 provides a detailed description of the implementation of the APTQ and APQ algorithms. Section 3 lists and evaluates the comparative experimental results. Finally, Section 4 summarizes the research presented in this paper.

## 2. Proposed Method

### 2.1. Global Power-of-Two Ternary Quantization Based on Unfixed Boundary Thresholds (APTQ)

#### 2.1.1. APTQ Quantization Strategy

According to Formulas (1)–(3), the conventional linear CNN quantization process based on fixed boundary thresholds and intervals can be approximated as

(4) w ≈ Sq,

where *w* represents the original floating-point weights to be quantized, *q* represents the corresponding quantized fixed-point weights, and S represents the quantization scale factor, which is calculated using Formula (1). In order to calculate *S*, it is necessary to determine the actual range of the floating-point weight value corresponding to the quantization value. Therefore, it can be said that the function of *S* is to divide a given quantization area into several intervals. After selecting the intervals and the corresponding floating-point boundary thresholds $\theta_{i}$, the floating-point weights in each range can be approximated to the corresponding fixed-point number. Previous studies [40,64,65] have all quantized CNN models based on this foundation. However, only three quantization intervals are used in ternary quantization, which means that there are only two boundary values, $\theta_{1}$ and $\theta_{2}$, in a layer. These two values are equal in symmetric quantization, and, thus, the quantization process of conventional symmetric ternary quantization algorithms such as TWN and TTQ [40,41] can be defined as

(5) $$q_{i}=\left\{\begin{array}{ll} +1, & \mathrm{if}W_{l}\;>\;\theta \\ 0, & \mathrm{if}\left|W_{l}\right|\leq \theta \\ -1, & \mathrm{if}W_{l}<-\theta \end{array}\right.,$$

where $W_{l}$ represents the set of weights to be quantized in each CNN layer, and *θ* and −*θ* represent the fixed boundary thresholds, which split the floating-point weights into {−1, 0, +1}. Both algorithms use the Euclidean distance as the standard to measure the quantization performance, and the whole ternary quantization process can be summarized as an optimization problem as follows:

(6) $$\left\{\begin{array}{c} S^{*},\;q^{*}=\arg\underset{S,q}{\min}J(S,\;q)={\left\|w-\mathrm{Sq}\right\|}_{2}^{2}\\ \&s.t.S\geq 0,q\in \{-1,\;0,\;+1\} \end{array}\right.,$$

where arg min (·) indicates the value of the variable when the objective function takes the minimum value. ${\left\|\cdot \right\|}_{2}^{2}$ represents the square of the Euclidean distance, and *w*, *S*, and *q* are defined as in Formula (4). As the algorithm cannot directly determine the boundary threshold *θ*, TWN defines *θ* as

(7) $$\theta \;=0.7\cdot (\left|w\right|)\;\approx \;\frac{0.7}{N}\sum_{i=1}^{N}\left|w_{i}\right|,$$

where *N* represents the number of weights. It can be seen that *θ* here is a fixed boundary threshold. In our previous research on power-of-two quantization, we also adopted the method of customizing the fixed floating-point boundary thresholds according to the range of floating-point values in each CNN layer to complete quantization [46]. The essence of most quantization studies is to seek several of the best floating-point boundary thresholds $\theta_{i}$ in the quantization process and to quantize the weights in the fixed region to their corresponding $\theta_{i}$ values. These fixed boundary thresholds will become the primary constraint factor for the CNN ternary quantization performance due to the uneven weight distribution, especially in the case of extremely low bit quantization. Based on the TWN and GSNQ algorithms, this paper proposes a global power-of-two ternary quantization algorithm based on an unfixed boundary threshold, APTQ, to remove these restrictions.

We introduce the quantization strategy and the complete quantization process of the APTQ algorithm through the ternary quantization of convolutional layers. As the fully connected layers in CNNs can also be expressed in the form of convolution calculations, the quantization method for fully connected layers is the same as that for convolutional layers. Assuming a floating-point CNN model with a total of *L* layers $\{W_{l}:\;1\;\leq \;l\;\leq \;L\},$ the weight set in the *l*th convolutional layer is expressed as $W_{l},$ including a total number of *i* floating-point filters $F_{i}$. The proposed APTQ algorithm requires initially dividing the network to be quantized into two structurally identical subnetworks. Specifically, for individual filters, all the filters $F_{i}$ to be quantized are correspondingly split into two subfilters, $F_{i1}$ and $F_{i2\;}$. These filters and their weights simultaneously satisfy the following formulas:

(8) $$F_{i}=F_{i1}+F_{i2},$$

(9) $$\left\{\begin{array}{c} a=a_{1}+a_{2}\\ s.t.\left|a_{1}\right|,\left|a_{2}\right|<\min\{\max(W_{l}),\left|\min(W_{l})\right|\} \end{array},\right.$$

where *a*, $a_{1}$, and $a_{2}$ refer to the corresponding floating-point weight values in the same position in $F_{i}I,$ $F_{i1}$, and $F_{i2},$ respectively; max (·) means to take the maximum value; and min (·) means to take the minimum value. The restriction on $a_{1}$ and $a_{2}$ in Formula (9) is very important. Without this restriction, large errors can easily occur, thereby resulting in a large accuracy loss. Subsequently, the two subnetworks undergo binary quantization, and $F_{i1}$ and $F_{i2}$ are optimized for binary quantization according to Formula (6) to obtain two binary fixed-point filters, $B_{i1}$ and $B_{i2\;}$, respectively, the weights of which belong to {−1, 1}. If one wishes to recombine the two binary subnetworks into a new ternary quantized network, it is necessary to ensure that the weights in the two binary matrices derived from the same original matrix have the same absolute values, i.e., the quantization scale factor *S* is the same, to ensure that their sum can be represented in ternary form. Thus, the binary quantization process for the two subnetworks can be expressed as

(10) $$\left\{\begin{array}{c} J(S_{i1},S_{i2},B_{i1},B_{i2})={\;\left\|F_{i1}-S_{i1}B_{i1}\right\|}_{2}^{2}+{\left\|F_{i2\;}-S_{i2}B_{i2}\right\|}_{2}^{2}\\ s.t.S_{i1}=S_{i2}=S_{i};S_{i}\;\geq \;0 \end{array}\right.,$$

where $S_{i1}$ and $S_{i2}$ represent the quantization scale factors of $B_{i1}$ and $B_{i2},$ respectively, which can be uniformly denoted as $S_{i\;}$, and J (·) represents the objective to be obtained in this optimization problem. Although each binary quantization process is also based on a fixed boundary threshold quantization method, the combined quantized values can allow certain weights to exceed the constraints of the boundary thresholds. Formula (10) can be expanded to obtain the values as follows:

(11) $$J\;(S_{i1},S_{i2},\;B_{i1},\;B_{i2})=S^{2}(B_{i1}^{T}B_{i1}+B_{i2}^{T}B_{i2})\;-2S(B_{i1}^{T}F_{i1}+B_{i2}^{T}F_{i2})+(F_{i1}^{T}F_{i1}+F_{i2}^{T}F_{i2}).$$

As $F_{i1}$ and $F_{i2}$ are known, $F_{i1}^{T}F_{i1}+F_{i2}^{T}F_{i2}$ and $B_{i1}^{T}B_{i1}+B_{i2}^{T}B_{i2}$ are known constants. In order to bring the spatial distribution of the ternary convolutional layer closest to that before quantization, it is necessary to minimize the sum of squares of the Euclidean distance; then, $B_{i1}^{T}F_{i1}+B_{i2}^{T}F_{i2}$ needs to keep the maximum value. When $B_{i1}$ and the corresponding $F_{i1}$ have the same sign at the same position, the largest $B_{i1}^{T}F_{i1}+B_{i2}^{T}F_{i2}$ can be obtained. Thus, the solution formulas are as follows:

(12) $$B_{i1}^{*}=\mathrm{sgn}\;(F_{i1}),$$

(13) $$B_{i2}^{*}=\mathrm{sgn}\;(F_{i2}),$$

where sgn (·) represents the sign function. According to the obtained $B_{i1}^{*}$ and $B_{i2}^{*},$ the optimal quantization scale factor $S_{i}^{*}$ can also be calculated using the following formula:

(14) $$S_{i}^{*}=\frac{\sum_{k=1}^{N}\left|F_{i1k}\right|+\sum_{k=1}^{N}\left|F_{i2k}\right|}{2N},$$

where $F_{i1k}$ and $F_{i2k}$ represent the elements in filters $F_{i1}$ and $F_{i2\;}$, respectively, and *N* represents the total number of weights in this convolutional layer, which means that all filters in the entire convolutional layer will have the same optimal scaling factor. At this time, we can define the part $T_{i}$ that completes the ternary quantization as in Formula (15). This completes ternary quantization with unfixed boundary thresholds, thereby allowing for higher quantization accuracy than fixed boundary threshold ternary quantization.

(15) $$T_{i}={S_{i}^{*}B}_{i1}+{S_{i}^{*}B}_{i2}.$$

However, the ternary quantization result obtained using Formula (15) is the same as that of the TTQ algorithm [41], which quantizes the weights in each layer to 0 and two other positive and negative 32-bit floating-point values. Although this can result in better software performance, it will complicate the implementation of the original CNN model when deploying to embedded chips in edge computing application devices. It still requires considerable on-chip computation and storage resources, and the computing efficiency is low, thereby resulting in poor practicability and hardware-unfriendly characteristics. To achieve hardware-friendly power-of-two quantization, after calculating the optimal $S_{i}^{*}$ using Formula (14), this paper further approximates $S_{i}^{*}$ to the nearest power of two to obtain the final quantization scale factor $S_{i}^{**}$:

(16) $$\left\{\begin{array}{c} sub=\min(\left|\mathrm{list}(p)\;-S_{i}^{*}\right|)\\ s.t.\mathrm{list}(p)=\{1,0.5,0.25,0.125,\ldots \} \end{array}\right.,$$

(17) $$S_{i}^{**}=\mathrm{return}\;\left[\mathrm{list}(p)\right]\to \mathrm{sub},$$

where list(*p*) represents an array of positive power-of-two values arranged from largest to smallest starting from one. This is because the weights in a pretrained CNN model are generally less than one [52]. Furthermore, *sub* represents the absolute value of the minimum difference between the values in the list(*p*) arrays and $S_{i}^{*},$ and $S_{i}^{**}$ is the power-of-two value corresponding to *sub*. Finally, the ternary quantization is completed according to Formula (18):

(18) $$T_{i}={S_{i}^{**}B}_{i1}+{S_{i}^{**}B}_{i2}=S_{i}^{**}(B_{i1}+B_{i2})$$

Here, since both $B_{i1}$ and $B_{i2}$ belong to {−1, +1}, $T_{i}$ must be a power-of-two or 0 value, thereby completing the entire layer’s power-of-two ternary quantization.

**Figure 2:**
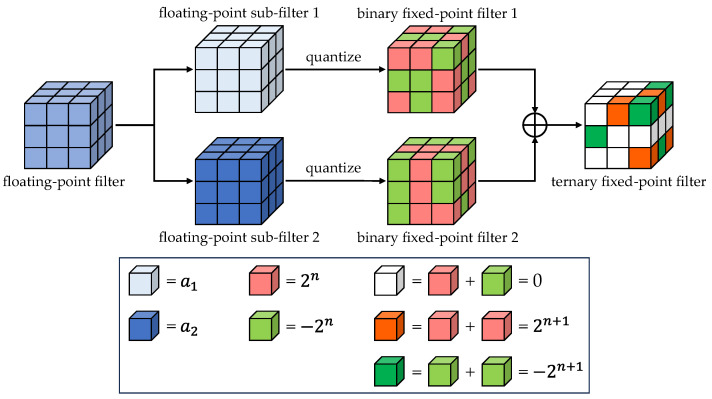
Strategy of power-of-two ternary quantization based on unfixed boundary thresholds.

In summary, the basis of the APTQ algorithm is to use Formulas (8), (9), (12)–(14), and (16)–(18) to decompose each filter into two subfilters, complete the binary quantization of the subfilters according to the obtained optimal power-of-two quantization scale factor, and finally add them together to resynthesize a power-of-two ternary filter with unfixed quantization boundary thresholds. Figure 2 takes a 3 × 3 × 3 filter as an example to intuitively illustrate the quantization process of the proposed APTQ algorithm.

#### 2.1.2. Weight Distribution Characteristics of APTQ Quantization Strategy

**Figure 3:**
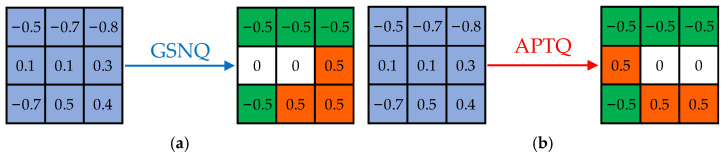
Quantization comparison of single convolution kernel based on fixed boundary thresholds and unfixed boundary thresholds: (**a**) schematic diagram of GSNQ quantization result and (**b**) schematic diagram of APTQ quantization result.

In the entire quantization process, due to the inherent randomness in decomposing floating-point filters into two subfilters, this is the fundamental reason why certain weights are released from the constraints of fixed quantization boundary thresholds. The final distribution of the quantized weights differs to some extent from conventional quantization algorithms. As illustrated in Figure 3, it compares the quantization results of a single convolutional kernel after power-of-two ternary quantization using the conventional quantization algorithm based on fixed quantization boundary thresholds and the APTQ algorithm proposed in this paper.

**Figure 4:**
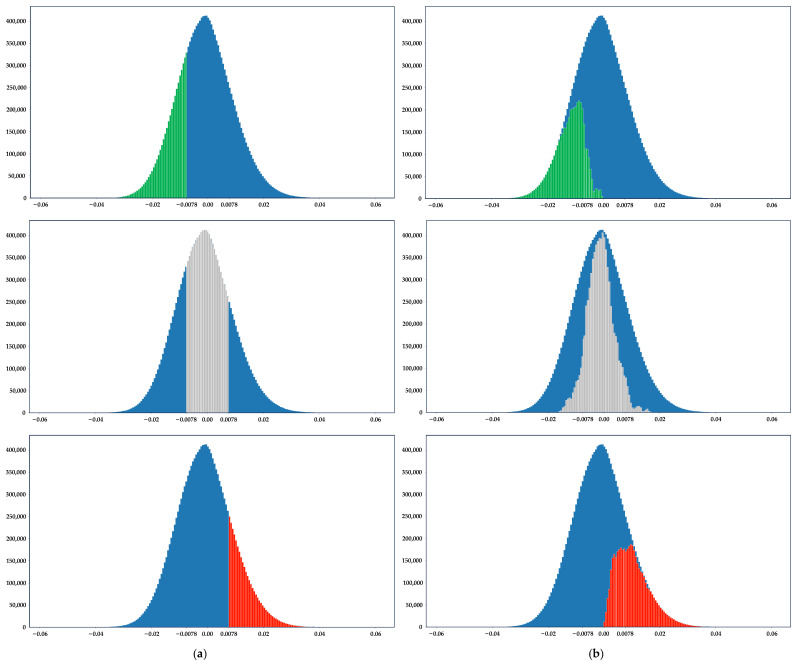
Comparison of quantization weight distribution between GSNQ and APTQ algorithms in a single convolutional layer: (**a**) ternary quantization based on fixed boundary thresholds and (**b**) ternary quantization based on unfixed boundary thresholds.

It can be seen from this example that, from the perspective of a single convolution kernel or filter, the APTQ algorithm can quantize a floating-point weight to a power-of-two value or 0 value with a farther L1 distance, such as quantizing 0.3 to a more distant 0, which seems unreasonable. And this is precisely the characteristic of the APTQ algorithm: breaking the inherent constraints of fixed quantization boundaries. While ensuring that the spatial Euclidean distance is minimized for the network layers and the entire network before and after quantization, it ensures that the CNN can be quantized more reasonably. This involves quantizing a portion of the weights within the same range to one quantized value and another portion to a different quantized value, thereby more effectively reducing the accuracy loss caused by low-bit-width quantization. Figure 4 shows a ternary quantization weight distribution comparison of the GSNQ and APTQ algorithms in the eighth convolutional layer of VGG-16.

The horizontal coordinates in the figure indicate the original floating-point weight values, and the vertical coordinates indicate the distribution number of the corresponding weights. The blue part indicates the overall weight distribution of the original floating-point weights in this convolutional layer. Both algorithms quantized all the weights in this layer to {${-2}^{-6}$, 0, $2^{-6}$}. In order to illustrate the difference between the quantization of the algorithm based on fixed thresholds and the algorithm based on unfixed thresholds more intuitively, we compared each of the three distributions of quantized values after employing quantization separately. The green, gray, and red parts indicate the distribution of the weights that were quantized to ${-2}^{-6}$, 0, and $2^{-6}$, respectively. As indicated by the gray area in Figure 3a, whereas the other quantization methods quantized all the weights to 0, the APTQ algorithm quantified a portion to ${-2}^{-6}$, another portion to $2^{-6}$, and another portion to 0. The same logic was applied to the green and red sections. This minimized the Euclidean distance for the entire network before and after quantization. This means that the quantization result of APTQ does not have fixed boundary thresholds, thereby breaking through the limitation of the fixed boundary threshold, which leads to a higher accuracy of the ternary CNN model.

#### 2.1.3. APTQ Global Retraining Process

**Figure 5:**
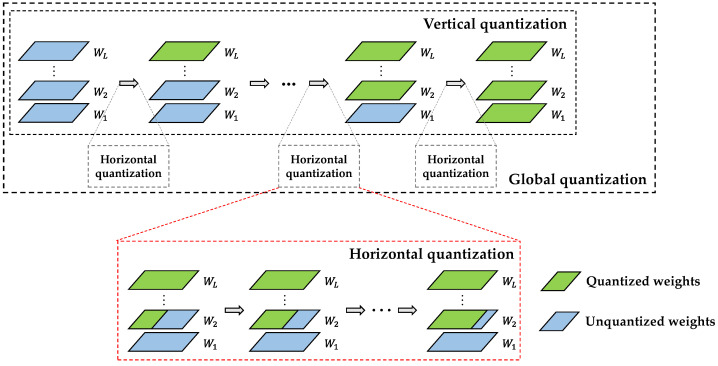
APTQ global fine-grained quantization architecture.

Regardless of the kind of quantization strategy, the expression ability of the values must decrease significantly when the weights change from 32-bit floating-point numbers to 2-bit fixed-point numbers, thus making some accuracy loss unavoidable. Therefore, it is necessary to retrain the quantization of the CNN models to compensate for the loss. The accuracy loss after one-time global quantization is often unrecoverable, and this paper proposes a fine-grained global quantization retraining architecture to maximally compensate for the low-bit quantization accuracy loss, which includes weights grouped by layer, layer-by-layer grouped quantization, and network retraining to gradually complete quantization of the entire CNN model. The specific process is shown in Figure 5.

This figure shows the whole quantization process of a CNN model with *L* layers. $W_{l}$ represents the weight set of the *l*th layer. Blue indicates unquantized portions, while green represents quantized portions. The whole architecture divides the CNN quantization process into two steps: horizontal (intralayer) and vertical (different layers) quantization. In the process of grouping weights by layer, the architecture takes filters as units, sorts the filters in each layer from large to small according to their L1 norm, and divides them into different numbers of groups (generally four groups per layer) according to the actual situation of different networks. A larger L1 norm means that this filter has a greater influence on the CNN model [27,52]. Therefore, in the subsequent quantization process, the filter group with a larger L1 norm is given priority in quantization. As each CNN layer needs to maintain the same three quantization values, for each layer, all of the filters in the same layer are split into two corresponding subfilters in the process of grouped quantization, as in Formulas (8) and (9), after which power-of-two ternary quantization is performed based on the unfixed boundary thresholds in the order of grouping. After quantizing a group, a CNN retraining process is performed to restore accuracy. In the retraining process, it is necessary to remerge the subfilters of the unquantized part in the layer where the current quantization group is located, keep all data in this layer and the previously quantized layer fixed during the SGD weight update process in backpropagation, and update all the unquantized weights to compensate for the accuracy loss caused by the quantized part. Then, in the quantization process of the next set, the remaining filters in this layer must be resplit into two parts (as in the first split) then quantized and retrained. Through continuous iteration, the whole CNN model can be finally quantized in a step-by-step manner, and the accuracy loss can be kept within an acceptable range as much as possible.

The process of the proposed APTQ algorithm is summarized in Algorithm 1:
**Algorithm 1:** Adaptive Global Power-of-two Ternary Quantization Based on Unfixed Boundary Thresholds (APTQ)**Input:** 32-bit floating-point CNN model$\;\{W_{l}:\;1\;\leq \;l\;\leq \;L\}$
**Output:** Power-of-two ternary quantization CNN model $\left\{Q_{l}:\;1\;\leq \;l\;\leq \;L\right\}$1: Grouping weights by layer: Sort filters by their L1 norm and divide them into M groups $\{D_{m}^{l}:\;1\;\leq \;m\;\leq \;M\}$2: **for** l∈ [1, …, L] **do**3:     Split all filters in the same layer into two subfilters using Formulas (8) and (9)4:     **for** m∈ [1, …, M] **do**5:      Determine the optimal quantization scale factor using Formulas (12)–(14), (16)      and (17), and complete power-of-two binary quantization of the two subfilters6:      Remerge the subfilters using Formula (18) to complete the power-of-two ternary      quantization based on unfixed boundary thresholds7:      Retrain the network, keep the quantized layers fixed, and update unquantized      weights in other layers8:     **end for**9:   **end for**

### 2.2. Universal Global Power-of-Two Quantization Based on Unfixed Boundary Thresholds (APQ)

The quantization strategy based on unfixed boundary thresholds proposed in this paper can relieve the limitation of quantization performance caused by the fixed quantization boundaries in ternary quantization, and it can effectively reduce the accuracy loss due to ternary quantization. At the same time, although weights with a higher bit width have superior expressive abilities and the quantization intervals are more tightly divided, many works have achieved higher accuracy results than the original CNN model with 4-bit and 5-bit quantization [43,46,59]. However, the limitation of fixed boundary thresholds with respect to the CNN quantization performance may still exist. In order to further improve the accuracy, this paper extends the APTQ algorithm mentioned in Section 2.1 to any bit width and proposes a new universal power-of-two quantization algorithm based on unfixed boundary thresholds, which is called APQ.

The main difference between *m*-bit and ternary quantization in the proposed quantization strategy lies in the number of subfilters divided by each filter and in the way the quantization scale factor is approximated to a power-of-two value. First, the APQ algorithm divides the filter $F_{i}$ in the first layer into $H={\;2}^{h}-\;2$ subfilters ${\{F}_{i1},\;F_{i2},\;\ldots ,\;F_{\mathrm{iH}}\}$, where h is the bit width to be quantized. At the same time, these subfilters need to satisfy the following conditions:

(19) $$F_{i}=F_{i1}+F_{i2}+\ldots +\;F_{\mathrm{iH}},$$

(20) $$\left\{\begin{array}{c} a=a_{1}+a_{2}+\ldots {+\;a}_{H}\\ s.t.\left|a_{1}\right|,\left|a_{2}\right|,\ldots ,\left|a_{H}\right|<\frac{1}{h-\;1}\min\{\max\left(W_{l}\right),\left|\min(W_{l})\right|\} \end{array}\right..$$

Similar to the APTQ algorithm, $a_{1}$ to $a_{H}$ here refer to the corresponding floating-point weights in the same position in terms of subfilters $F_{i1}$ to $F_{\mathrm{iM}}$, respectively. After the split is completed, we quantize all the divided subfilters based on the principle of minimizing Euclidean distance according to Formula (21):

(21) $$\left\{\begin{array}{c} J(S_{i1},\ldots ,S_{\mathrm{iH}};B_{i1},\ldots {,\;B}_{\mathrm{iH}})=\sum_{k\;=1}^{H}{\left\|F_{\mathrm{ik}}\;{–S}_{\mathrm{ik}}B_{\mathrm{ik}}\right\|}_{2}^{2}\\ s.t.S_{i1}=\ldots =S_{\mathrm{iH}\;}=S_{i};\;S_{i}\geq 0 \end{array}\right.,$$

In the APQ algorithm, $B_{\mathrm{iH}}^{*}$ also maintains the same sign at each corresponding position with the respective subfilter, and all quantized weights belong to {−1, +1}, as shown in Formula (22):

(22) $$B_{\mathrm{iH}}^{*}=\mathrm{sgn}(F_{\mathrm{iH}}),$$

The optimal quantization scale factor $S_{i}^{*}$ of the APQ algorithm changes based on the number of divided subfilters, which differs from the APTQ algorithm, as shown in Formula (23). When the quantization bit width *h* is 2-bit, it is equivalent to the $S_{i}^{*}$ of the APTQ algorithm:

(23) $$S_{i}^{*}=\frac{\sum_{j}^{H}\sum_{k\;=1}^{N}\left|F_{\mathrm{ik}}\right|}{\mathrm{HN}}.$$

Here, *N* represents the total number of weights in the convolutional layer, and *H* is the number of subfilters into which the original filter is divided.

As the APQ algorithm divides the filters into a variable number of subfilters, which may not necessarily be a multiple of two—such as 14 subfilters when quantized to 4-bit and 6 subfilters when quantized to 3-bit—if $S_{i}^{*}$ is directly approximated to a power-of-two value and then added together, the quantization result in the power-of-two form may not be obtained. Therefore, after obtaining $S_{i}^{*},$ in contrast to the APTQ algorithm, it is necessary to first remerge the binary subfilters and then approximate their weights to the nearest power-of-two value to achieve generalized power-of-two quantization based on unfixed boundary thresholds in the APQ. The specific process is shown in the Formulas (24)–(26):

(24) $$Q_{i}=S_{i}^{*}\sum_{k=1}^{M}B_{\mathrm{ik}},$$

(25) $$\left\{\begin{array}{c} sub=\min(\left|\mathrm{list}(p)\;-Q_{i}\right|)\\ s.t.\mathrm{list}(p)=\{1,0.5,0.25,0.125,\ldots \} \end{array}\right.,$$

(26) $$Q_{i}^{*}=\mathrm{return}\;\left[\mathrm{list}(p)\right]\to \mathrm{sub},$$

where $Q_{i}$ is the binary quantization filter obtained by remerging the subfilters, and $Q_{i}^{*}$ is the final quantized power-of-two result of $Q_{i}$ approximated to the nearest power-of-two value. The remaining quantization steps and retraining architecture remain the same as in the APTQ algorithm. The quantization process of the APQ algorithm can be summarized in Algorithm 2:
**Algorithm 2:** Universal Global Power-of-Two Quantization Based on Unfixed Boundary Thresholds (APQ)**Input:** 32-bit floating-point CNN model$\;\{W_{l}:\;1\;\leq l\leq L\}$
**Output:** Power-of-two *h*-bit quantization CNN model $\left\{Q_{l}:\;1\;\leq l\leq L\right\}$1: Grouping weights by layer: Sort filters by their L1 norm and divide them into M groups $\{D_{m}^{l}:\;1\;\leq \;m\;\leq \;M\}$2: **for** l∈ [1, …, L] **do**3:   Split all filters in the same layer into $H={\;2}^{h}-2$ subfilters using Formulas (19) and (20)4:   **for** m∈ [1, …, M] **do**5:     Determine the optimal quantization scale factor using Formulas (21)–(23), and    complete power-of-two binary quantization of the *H* subfilters6:     Merge the binary subfilters and approximate all values to the nearest power-of-two by    using Formulas (24)–(26), completing the *h*-bit power-of-two quantization of the original filter.7:     Retrain the network, keep the quantized layers fixed, and update unquantized    weights in other layers8:   **end for**9:  **end for**

### 2.3. APTQ and APQ Dedicated Convolutional Computation Module in Edge Chips

The two power-of-two quantization algorithms proposed in this paper quantize all the parameters in a CNN into power-of-two values. This characteristic enables the quantized model to be deployed in embedded chips for edge computing applications using simple shift operations instead of complex multiplication operations. Before deployment, the power-of-two weights need to be recoded into 2-bit binary numbers for storage in the edge chips as new weights for hardware calculations, as shown in Table 1 and Table 2.

**Figure 6:**
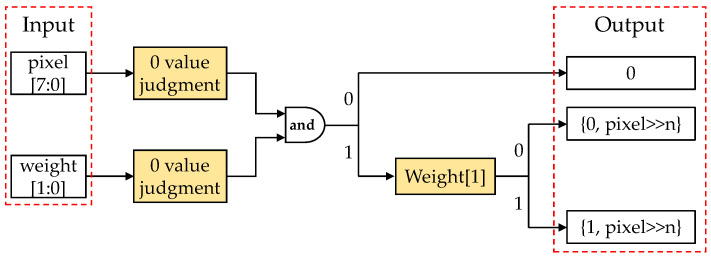
Dedicated multiplication processing unit for ternary quantization CNN models.

For the ternary CNN model quantized with APTQ, the chip determines whether the input binary-encoded weights and pixel values contain a zero value and identifies the sign of the quantized weights. It then performs a pre-set *n*-bit shift operation or directly outputs a zero value. Figure 6 shows the ternary quantization dedicated multiplication processing unit designed in an FPGA for the APTQ-quantized CNN model to perform the multiplication operation for one input pixel with one weight, thereby fully leveraging the advantages of ternary power-of-two quantization.

**Figure 7:**
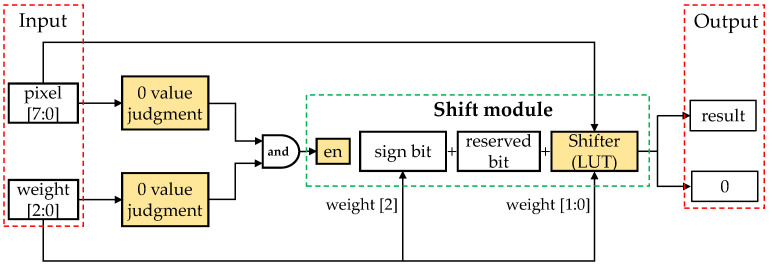
Dedicated multiplication processing unit for other bit width quantization CNN models.

For other bit width CNN models quantized using APQ, a more versatile shift-based multiplication processing unit was designed in this paper. As shown in Figure 7, it is a schematic diagram of a multiplication processing unit for a 4-bit quantized model. Each unit, through judgment modules, detects whether there are zero data in the input. An enable signal is generated by an AND gate, and when the enable signal is high, the unit performs shift-based calculations, thereby outputting results, including the sign bit and reserved bits. Otherwise, it directly outputs 0. This multiplication processing unit can be similarly adopted for models with other bit widths.

The specialized multiplication processing units designed for the above two approaches aim to leverage the advantages of APTQ and APQ quantization processes, thereby achieving zero occupation in the on-chip DSP computing units for edge chips. Utilizing this simple judgment and shift operation for multiplication in edge chips can be completed within three clock cycles, thereby minimizing on-chip computational resource utilization, enhancing computational efficiency, and facilitating the construction of a pipelined processing architecture.

**Figure 8:**
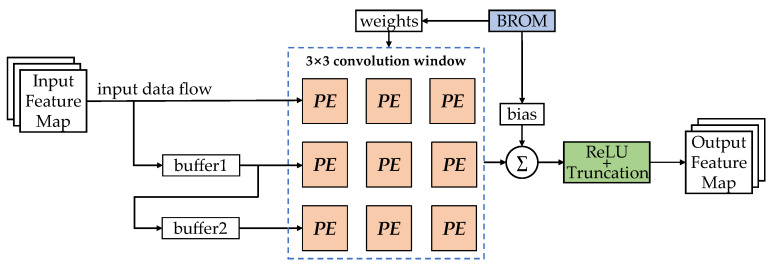
Convolutional computation module.

This structure can simplify the processing of multiplication operations in embedded chips such as FPGAs and ASICs. Furthermore, as the main computational complexity in CNNs derives from the convolutional computations in the convolutional layers, this means that in order to verify the effectiveness of APTQ for CNN hardware deployment, a complete convolutional computation architecture was also built for this study. The overall architecture adopted the same pipelined architecture as in Reference [46], as shown in Figure 8.

Due to the fact that only the forward inference process of CNN models needs to be completed in the embedded chip, the function of this computation module is to complete the convolutional computation of an input feature map with a 3 × 3 convolution kernel through an activation function, thereby finally obtaining an output feature map that can be used as an input for the next layer. *PE* here represents the multiplication processing unit shown in Figure 7 or Figure 8, and ∑represents a multiway accumulator. Through the design of multibuffer and convolution windows, the multiplexing of the input feature pixels can be realized, thereby forming an efficient pipeline computing mode. Furthermore, this convolution computation module can serve as a universal module for various CNNs. Changing the number of PEs in the convolution window and the corresponding number of buffers can realize the convolution computation for different convolution kernels sizes. Simultaneously, when on-chip hardware resources allow, multiple convolution computation modules can be parallelized to achieve convolution calculations for multichannel feature maps.

## 3. Experiments

In this section, we compare the proposed ternary quantization algorithm APTQ and the general quantization algorithm APQ with traditional ternary quantization and other bit width quantization algorithms in multisample and multiangle scenarios in order to verify the effectiveness and universality of the quantization strategy based on the unfixed quantization boundary thresholds proposed in this paper.

### 3.1. APTQ Quantization Performance Testing

#### 3.1.1. Implementation Details

Several commonly used convolutional neural network models, namely, AlexNet [66], VGG-16 [7], ResNet-18, ResNet-20, ResNet-56 [60] and GoogLeNet [61], were compared with APTQ on small sample classification data sets, including CIFAR10 [62], CIFAR100 [62], and Mini-ImageNet [51].

For the baseline CNN models, pretraining of the initial CNN models for 300 epochs in the data sets was completed, and the baseline models to be quantized for each CNN were obtained. The baseline models of the two CNNs ResNet-20 and ResNet-56 with respect to the CIFAR10 data set are given in Reference [60], which we directly tested. To enhance the processing of the data sets, we added a column or row of 0-value pixels to each side of the CIFAR10 and CIFAR100 images; we then performed random cropping and inversion. We completed the images in the Mini-ImageNet data set to a size of 256 × 256 and then performed random cropping and inversion. For the APTQ algorithm, the ternary quantization process followed the procedure shown in Algorithm 1. In the weight-grouping process, since the ResNet-20 and ResNet-56 models have a small number of parameters, a slight change will also have a large impact on their precision performance, so we divided the filters in each layer of these two networks into five groups and quantized them in a group-by-group manner. For the other four networks, due to their larger model size and larger number of parameters, thus resulting in stronger resistance to interference, we divided the filters in each layer into four groups and then quantized them group by group. During the process of CNN retraining, 20 epoch updates were set in each retraining process to fully recover the accuracy loss. The other important training hyperparameters were basically consistent with those mentioned in the GSNQ algorithm [46], as shown in Table 3.

All quantization performance testing experiments in this paper were conducted using Pycharm Community Edition 2022.1.1 as the development environment with software coding based on the PyTorch library [67], and they were performed on an Nvidia GeForce RTX 3070Ti GPU (Santa Clara, CA, USA).

#### 3.1.2. APTQ Quantization Performance Comparison

Quantization research was first conducted on multiple samples using the proposed APTQ algorithm and the GSNQ algorithm proposed in our previous work on different CNN models and data sets. As a global power-of-two quantization algorithm, the GSNQ algorithm already has better quantization performance than most existing algorithms at 3-bit and above, but it performs poorly in the case of ternary quantization (2-bit), with accuracy losses exceeding 1.5% in many cases. The APTQ algorithm’s ternary quantization strategy based on unfixed boundary thresholds is designed to address this issue, and it adopts a global quantization architecture similar to the GSNQ algorithm while changing the quantization strategy. Therefore, comparing the APTQ algorithm with the GSNQ algorithm is the most intuitive way to verify the effectiveness of the quantization strategy based on unfixed quantization boundary thresholds. Table 4, Table 5 and Table 6 provide the comparison results regarding the quantization accuracy between the two algorithms for various networks with respect to the CIFAR10, CIFAR100, and Mini-ImageNet data sets, respectively, in order to verify whether the APTQ algorithm can generally and effectively improve the CNN ternary quantization performance. Among them, bold represents the result of the algorithm in this paper.

Based on the comparison results, it can be seen that, in the different data sets and CNN models, the ternary quantization results of the GSNQ and APTQ algorithms both presented a certain accuracy loss when compared to the baseline models. However, the accuracy loss of the APTQ algorithm was smaller than that of the GSNQ algorithm in all cases, and the APTQ algorithm maintained the loss of the top-1 accuracy within 1%. For example, ResNet-56 in the CIFAR10 data set showed an astonishing accuracy loss of only 0.13% after APTQ ternary quantization, thereby achieving almost zero accuracy loss quantization. These two sets of experiments fully verify that the quantization strategy based on unfixed boundary thresholds in APTQ plays a significant role in ternary quantization, thus presenting strong universality.

In order to further verify the performance of APTQ ternary quantization, it was compared with some other state-of-the-art ternary quantization algorithms in the literature, including TWN [40], DSQ [44], LQ-Net [68], DoReFa-Net [69], PACT [39], ProxQuant [70], APoT [59], and CSQ [71]. Among them, the APoT algorithm is also a power-of-two quantization algorithm, while the other algorithms are conventional ternary algorithms. The results are shown in Table 7.

The results demonstrate that the existing state-of-the-art quantization algorithms were all basically unable to achieve ternary quantization without resulting in accuracy loss. However, the performance of the proposed APTQ algorithm was better than that of most other methods and was only slightly lower than some methods in rare cases, such as the CSQ algorithm for ResNet-20. On the other hand, among the power-of-two ternary quantization algorithms (APTQ, GSNQ, and APoT), APTQ presented the best quantization performance. APTQ implements global quantization, while most other methods still retain some weights that are not quantized or 8-bit quantized.

The above experiments were all direct quantitative tests of quantization accuracy, which were conducted in order to intuitively analyze the software performance of APTQ. We also calculated the sum of the L2 distances for all filters in the whole CNN model between the ternary models quantized by some different algorithms and their original baseline models in order to qualitatively analyze the performance of APTQ. The results are shown in Table 8. It can be seen that, among the five methods of TWN, LQ-Net, APoT, GSNQ, and APTQ, the L2 distance of the APTQ method was the shortest, which indicates that the ternary CNN model developed with APTQ had the smallest gap with respect to the baseline model and was the most similar in terms of space. This result proves that the APTQ algorithm theoretically has the best performance.

In summary, a substantial amount of quantitative and qualitative comparative analysis has demonstrated the superior quantization performance of the APTQ algorithm at the software level compared to the majority of existing ternary quantization algorithms. The quantization strategy based on nonfixed boundary thresholds does indeed universally and effectively reduce the precision loss caused by fixed boundary thresholds during ternary quantization.

### 3.2. APQ Quantization Performance Testing

The implementation details of the APQ algorithm are the same as those of the APTQ algorithm. As 5-bit bit quantization and above basically achieved an accuracy equal to or even higher than the CNN baseline model in previous studies [33,59,71,72]. This section mainly focuses on quantization comparison experiments of the APQ algorithm with other state-of-the-art algorithms with respect to the CIFAR10 data set for 4-bit (15 quantization values per layer) and 3-bit (seven quantization values per layer) scenarios. The results are shown in Table 9, where the quantization accuracy values of the DoReFa-Net, PACT, LQ-Net, APoT, and GSNQ algorithms were taken from their corresponding studies.

The results in the table indicate that the performance of APQ at 4-bit and 3-bit quantization was almost the same as that of the APoT and GSNQ algorithms, with a small increase in accuracy, but it was higher than several other algorithms. Significantly, the 4-bit quantization performance of APQ showed a small increase compared to its 3-bit quantization performance, while the 5-bit quantization performance showed an even smaller increase compared to 4-bit. Therefore, upon synthesizing the experimental results considering APTQ ternary quantization and APQ universal quantization, it can be recognized that the approach for quantization based on unfixed quantization boundary thresholds proposed in this paper is more suitable for the quantization case of very low bit widths (e.g., ternary quantization), and it can greatly optimize the quantization performance. However, when considering higher quantization bit widths with more quantization values and stronger weight expression ability, it has little impact on the quantization results, and the enhancement effect is not obvious. However, although the 2-bit quantization performance of APTQ is better than that of most existing algorithms, when compared with quantization with higher bit widths, the performance degradation caused by less-reserved information at low bit widths is still inevitable. Therefore, in practical applications, it is necessary to weigh the quantitative performance according to the requirements of the corresponding scenario. If the edge application scenario is sensitive to latency, it is necessary to sacrifice some CNN accuracy performance and choose quantization with low bit width to reduce inference time. Conversely, quantization ranging from 4-bit to 8-bit can be chosen to ensure CNN model performance. For scenarios that require low latency and high precision, such as assisted driving, 3-bit or 4-bit quantization can be considered.

### 3.3. Hardware Performance Evaluation

#### 3.3.1. Implementation Details

After completing the quantization of APTQ and APQ on the GPU, this paper conducted preliminary deployment of the quantized power-of-two models on an FPGA platform. A comparison of the hardware resource utilization of the dedicated modules designed in Figure 6 and Figure 7 was performed to validate the assistance of the APTQ and APQ algorithms for the edge deployment of CNNs. All performance experiments related to hardware deployment used Vivado Design Suite—HLx Editions 2019.2 as the development environment, which were written in the Verilog hardware description language and compiled in the programmable logic side (FPGA side) of a ZYNQ XC7Z035FFG676-2I chip (Xilinx, San Jose, CA, USA). This chip contains 171,900 on-chip lookup tables (LUTs), 343,800 on-chip flip-flops (FFs), and 900 on-chip DSPs.

#### 3.3.2. Hardware Design Comparative Testing

The hardware advantage of the APTQ and APQ quantization algorithm is most intuitively reflected in terms of model storage space. Table 10 shows the comparison of storage space occupied by the CNN models before and after quantization.

It can be seen that, after encoding the APTQ quantization model using the method shown in Table 1, the size of the model deployed to the chip was significantly reduced. Models that do not exceed 10 Mb can be easily stored in the on-chip memory of the chip, thereby fundamentally avoiding the decrease in computing efficiency and energy consumption caused by frequent access to external memory by the chip.

Based on the architecture mentioned in Section 2.2, the *PE*s were changed to shift-based *PE*s for the ternary power-of-two quantization (see Figure 6 based on APTQ) designed in this paper, shift-based *PE*s for the 3-bit power-of-two quantization (see Figure 7 based on APQ) designed in this paper, *PE*s using on-chip DSP to implement multiplication calculations [15], and *PE*s based on the method provided by the Xilinx official documentation for implementing multiplication computation using lookup tables (LUTs) [73], respectively. And then, the on-chip hardware resource occupancy of the four modules on the FPGA platform was compared, as shown in Table 11.

Module 1 in the table represents the most common design for convolutional calculation [15,74], where the on-chip DSP resource usage depends on the number of processing elements (PEs) within the module. The scarcity of on-chip DSP resources hindered the parallel processing capability of the CNNs in the edge chips. Module 2 utilizes LUTs for multiplication through the Vivado 2019.2 compilation software, thereby relieving the restrictions imposed by the on-chip DSP. However, as a trade-off, there was a significant increase in the consumption of other on-chip resources. In contrast, Modules 3 and 4, which were designed based on the quadratic quantization characteristics of APTQ and APQ, achieved zero DSP resource utilization. Moreover, compared to Modules 1 and 2, these modules effectively reduced the total occupancy of all other on-chip hardware resources. Module 4, in comparison to Module 3, demonstrates a 2-bit model (ternary) that can further reduce resource usage by 1/3 compared to the 3-bit model, thereby offering an optimal saving of computational resources in embedded chips.

In most cases, the number of input and output channels for the convolutional layers in the convolutional neural networks ranged between 32 and 512 and was always maintained as a multiple of 32. Therefore, in FPGA deployment, regardless of the method used to accelerate convolutional neural networks, it is necessary to construct an architecture for a convolution computation accelerator with at least 32 parallel convolution acceleration modules, which serve as the fundamental processing units for convolutional layer computations [75,76]. In this paper, the designed convolution acceleration module was also subjected to 32-way parallel processing. Four acceleration modules were compared to validate the performance of the proposed quantization algorithm and dedicated computation modules in practical applications. Table 12 presents the hardware resource consumption of the different convolution acceleration modules for a 3 × 3 convolution kernel in the case of 32-way parallel processing.

After processing in a 32-way parallel manner, the utilization situation of different hardware resources became more pronounced. It is evident that the on-chip DSP resources were heavily consumed, while other hardware resources experienced less consumption. If, on top of these 32 parallel processing units, further parallel stacking is applied, the on-chip DSP resources on the XC7Z035 chip may become insufficient, thereby becoming a limiting factor for network deployment. This necessitates the use of FPGA chips with more on-chip resources, thereby leading to an inevitable increase in costs. Similar to the comparison results with a single convolution computation module, the two proposed quadratic algorithms in this paper effectively address the limitations imposed by on-chip DSP resources on CNN edge deployment performance. Additionally, the APTQ algorithm maximally assists CNNs in increasing their parallel processing capability (theoretically having the potential to achieve nearly 2.5 times the computational parallelism compared to the current most commonly used 8-bit model deployment), thereby enhancing the computational efficiency of edge deployment. The computational advantage, coupled with the significant reduction in storage volume from low-bit-width quantization and the high-precision advantages of the quantized network, enables the APTQ algorithm to balance the performance of quantized convolutional neural networks in terms of both software and hardware. As a result, it can be applied to a wide range of edge computing scenarios, thereby demonstrating strong industrial application value.

## 4. Limitation Discussion

The APTQ and APQ algorithms proposed in this paper effectively balance the software quantization performance of CNNs and hardware deployment efficiency. Experiments on various datasets and CNN models have validated their robust performance. However, to ensure the precision of low-bit quantization, this paper employed a fine-grained global retraining architecture during the quantization process. While this architecture minimizes precision loss through fine-grained group quantization and retraining, it introduces significant time costs and imposes higher demands on GPU computational power. When dealing with extremely large datasets such as ImageNet, a GPU with substantial computational power is required to complete the entire quantization process, thereby potentially also incurring significant time costs. The iterative network retraining process constitutes a major contributor to the extensive time costs. Without increasing the GPU computational power, it may be possible to reduce this limitation in training time costs through methods like learning rate rewinding [77], thereby making the proposed algorithm applicable to a wider range of scenarios.

## 5. Conclusions

This paper proposed an innovative power-of-two quantization method based on unfixed quantization boundary thresholds, called APTQ, in order to address the storage and computational pressure of CNNs on hardware that exists in edge applications through quantization at extremely low bit widths. APTQ employs a fine-grained quantization architecture with three-step iterations of filter grouping by layer, group quantization, and network retraining. In the quantization process, the optimal power-of-two quantization scale factor is calculated adaptively, according to the weight distribution of each layer, and each filter is quantized into the form of combining two power-of-two binary subfilters such that there is no need to set fixed boundary thresholds. In this way, a power-of-two ternary network model is adaptively obtained. It is worth noting that the APTQ algorithm not only performs well in terms of its hardware-friendly characteristics, but it also achieved excellent quantization performance for CNN models commonly used in industrial applications. The ternary quantization experiments on a variety of CNN models fully validate that the accuracy loss of APTQ can be maintained within 0.5% in most cases, thereby possessing almost state-of-the-art ternary quantization performance. By extending the quantization strategy of APTQ to any bit width, the proposed universal APQ quantization algorithm also has good quantization performance, thereby providing a choice for a wider range of application scenarios. In the next phase of our work, we intend to first optimize the performance of APQ in the context of high-bit-width quantization, and then we intend to extend the APTQ and APQ algorithms to other object detection models (e.g., YOLO and R-CNN), explore their adaptability to most CNN models, and further optimize and promote the standardization of the algorithm. Finally, we hope to fully deploy the quantified low-bit CNN model on an FPGA to complete the entire process of CNN edge deployment.

In conclusion, the APTQ and APQ algorithms can provide excellent quantization accuracy performance, less bandwidth pressure, lower storage and computational pressure, and higher computing efficiency of convolutional neural networks during deployment in embedded hardware. The two methods have good reference value for the field of industrial edge computing, and they can provide feasible and effective solutions for the efficient deployment of convolutional neural networks in edge devices.

## Figures and Tables

**Table 1:** Hardware deployment recoding of APTQ ternary quantized weights.

Quantized Weight	Recoding Weight
$2^{n}$	01
0	00
${-2}^{n}$	11

**Table 2:** Hardware deployment recoding of APQ 3-bit quantized weights.

Quantized Weight	Recoding Weight
$2^{n+2}$	001
$2^{n+1}$	010
$2^{n}$	011
0	000
${-2}^{n}$	111
${-2}^{n+1}$	110
${-2}^{n+2}$	101

**Table 3:** Quantization retraining hyperparameters for various CNN models.

CNN	Weight Decay	Momentum	Learning Rate	Batch Size
AlexNet	0.0005	0.9	0.01	256
VGG-16	0.0005	0.9	0.01	128
ResNet-18	0.0005	0.9	0.01	128
ResNet-20	0.0001	0.9	0.1	256
ResNet-56	0.0001	0.9	0.1	128
GoogLeNet	0.0002	0.9	0.01	128

**Table 4:** Ternary quantization results for the CIFAR10 data set.

CNN	Method	Top-1 Accuracy	Top-5 Accuracy	Decrease in Top-1/Top-5 Error
AlexNet	Baseline	82.96%	99.09%	
	GSNQ	80.95%	98.71%	−2.01%/−0.38%
	**APTQ**	**82.25%**	**99.01%**	**−0.71%/−0.08%**
VGG-16	Baseline	88.74%	99.59%	
	GSNQ	87.14%	99.28%	−1.60%/−0.31%
	**APTQ**	**88.18%**	**99.46%**	**−0.56%/−0.13%**
ResNet-18	Baseline	89.72%	99.69%	
	GSNQ	88.91%	99.40%	−0.81%/−0.29%
	**APTQ**	**89.20%**	**99.60%**	**−0.52%/−0.09%**
ResNet-20	Baseline	91.60%	99.76%	
	GSNQ	90.91%	99.61%	−0.69%/−0.15%
	**APTQ**	**91.21%**	**99.66%**	**−0.39%/−0.10%**
ResNet-56	Baseline	93.20%	99.80%	
	GSNQ	92.92%	99.69%	−0.28%/−0.11%
	**APTQ**	**93.07%**	**99.74%**	**−0.13%/−0.06%**
GoogLeNet	Baseline	90.04%	99.91%	
	GSNQ	89.02%	99.66%	−1.02%/−0.25%
	**APTQ**	**89.63%**	**99.75%**	**−0.41%/−0.16%**

**Table 5:** Ternary quantization results for the CIFAR100 data set.

CNN	Method	Top-1 Accuracy	Top-5 Accuracy	Decrease in Top-1/Top-5 Error
AlexNet	Baseline	70.11%	88.18%	
	GSNQ	67.12%	87.77%	−2.99%/−0.41%
	**APTQ**	**68.99%**	**88.18%**	**−1.12%/0.00%**
VGG-16	Baseline	72.03%	91.25%	
	GSNQ	70.00%	90.85%	−2.03%/−0.40%
	**APTQ**	**71.11%**	**91.10%**	**−0.92%/−0.15%**
ResNet-18	Baseline	74.16%	91.96%	
	GSNQ	73.17%	91.40%	−0.99%/−0.56%
	**APTQ**	**73.79%**	**91.71%**	**−0.37%/−0.25%**
GoogLeNet	Baseline	76.68%	92.01%	
	GSNQ	75.52%	91.58%	−1.16%/−0.43%
	**APTQ**	**75.98%**	**91.95%**	**−0.70%/−0.06%**

**Table 6:** Ternary quantization results for the Mini-ImageNet data set.

CNN	Method	Top-1 Accuracy	Top-5 Accuracy	Decrease in Top-1/Top-5 Error
AlexNet	Baseline	61.21%	86.99%	
	GSNQ	59.03%	84.65%	−2.18%/−0.51%
	**APTQ**	**60.49%**	**84.99%**	**−0.72%/−0.20%**
VGG-16	Baseline	75.09%	91.56%	
	GSNQ	73.51%	90.01%	−1.58%/−1.55%
	**APTQ**	**74.66%**	**90.52%**	**−0.43%/−1.04%**
ResNet-18	Baseline	76.76%	92.16%	
	GSNQ	75.19%	91.06%	−1.57%/−1.10%
	**APTQ**	**75.98%**	**92.00%**	**−0.78%/−0.16%**
GoogLeNet	Baseline	78.91%	93.10%	
	GSNQ	77.89%	92.61%	−1.02%/−0.49%
	**APTQ**	**78.29%**	**92.96%**	**−0.62%/−0.14%**

**Table 7:** Comparison with other ternary quantization algorithms on the CIFAR10 data set.

CNN	Method	Top-1 Accuracy	Decrease in Top-1 Error
VGG-16	Baseline	88.74%	
	TWN	86.19%	−2.55%
	DSQ	88.09%	−0.65%
	LQ-Net	88.00%	−0.74%
	**APTQ**	**88.18%**	**−0.56%**
ResNet-18	Baseline	89.72%	
	TWN	87.11%	−2.61%
	LQ-Net	87.16%	−2.56%
	DSQ	89.25%	−0.47%
	**APTQ**	**89.20%**	**−0.52%**
ResNet-20	Baseline	91.60%	
	DoReFa-Net	88.20%	−3.40%
	PACT	89.70%	−1.90%
	LQ-Net	90.20%	−1.40%
	ProxQuant	90.06%	−1.54%
	APoT	91.00%	−0.60%
	CSQ	91.22%	−0.38%
	**APTQ**	**91.21%**	**−0.39%**
ResNet-56	Baseline	93.20%	
	PACT	92.50%	−0.70%
	APoT	92.90%	−0.30%
	**APTQ**	**93.07%**	**−0.13%**

**Table 8:** The sum of ResNet-20 L2 distance before and after ternary quantization.

ResNet-20	TWN	LQ-Net	APoT	GSNQ	APTQ
L2 Distance	12.15	11.30	11.31	10.65	9.38

**Table 9:** Comparison of APQ with other quantization algorithms with respect to the CIFAR10 data set.

CNN	Method	Top-1 Accuracy		
		5-bit	4-bit	3-bit
ResNet-20 (baseline: 91.60%)	DoReFa-Net	—	90.5%	89.9%
	PACT	—	91.7%	91.1%
	LQ-Net	—	—	91.6%
	APoT	—	92.3%	92.2%
	GSNQ	—	92.42%	91.96%
	**APQ**	**92.42%**	**92.36%**	**92.16%**
ResNet-56 (baseline: 93.20%)	APoT	—	94.0%	93.9%
	GSNQ	—	94.0%	93.62%
	**APQ**	**93.99%**	**93.88%**	**93.67%**

**Table 10:** APTQ and APQ quantization model storage space comparison.

CNNs	CNN Models Storage Space		
	Baseline	After APQ (3-Bit)	After APTQ (2-Bit)
VGG-16	114.4 Mb	10.7 Mb	7.2 Mb
ResNet-20	4.5 Mb	0.4 Mb	0.3 Mb
ResNet-56	14.2 Mb	1.3 Mb	0.9 Mb

**Table 11:** The hardware resource occupancy of a single 3 × 3 convolution calculation module.

Modules	Bit Width	LUT	FF	DSP
Module 1: based on multiplication (implemented using on-chip DSP)	8-bit	428	392	9
Module 1: based on multiplication (implemented using on-chip DSP)	3-bit	250	268	9
Module 2: based on multiplication (implemented using on-chip LUT)	3-bit	402	226	0
Module 3: based on APQ	3-bit	263	237	0
Module 1: based on multiplication (implemented using on-chip DSP)	2-bit	158	191	9
Module 2: based on multiplication (implemented using on-chip LUT)	2-bit	262	158	0
Module 4: based on APTQ	2-bit	168	167	0

**Table 12:** The hardware resource occupancy of a 32-way 3 × 3 convolution calculation module.

Modules	Bit Width	LUT	FF	DSP
Module 1: based on multiplication (implemented using on-chip DSP)	8-bit	14,548/8.46%	11,226/3.27%	288/32.00%
Module 1: based on multiplication (implemented using on-chip DSP)	3-bit	8675/5.05%	9332/2.71%	288/32.00%
Module 2: based on multiplication (implemented using on-chip LUT)	3-bit	14,028/8.16%	7901/2.30%	0/0.00%
Module 3: based on APQ	3-bit	8510/4.95%	8270/2.41%	0/0.00%
Module 1: based on multiplication (implemented using on-chip DSP)	2-bit	5243/3.05%	6479/1.88%	288/32.00%
Module 2: based on multiplication (implemented using on-chip LUT)	2-bit	8475/4.93%	5370/1.56%	0/0.00%
Module 4: based on APTQ	2-bit	5862/3.41%	5799/1.69%	0/0.00%

## Data Availability

No new data were created or analyzed in this study. Data sharing is not applicable to this article.
